# Supplementary material for: HIVprotI: an integrated web based platform for prediction and design of HIV proteins inhibitors
Source: J Cheminform. 2018 Mar 9;10:12. doi: 10.1186/s13321-018-0266-y (PMC5845081; doi:10.1186/s13321-018-0266-y)
Supplement: Supplementary file 2 — Additional file 2. Source code of HIVProtI web server. [file 13321_2018_266_MOESM2_ESM.zip › HIVprotI_Source-code/hivprotI/weka-3-6-6/doc/weka/attributeSelection/StartSetHandler.html]

StartSetHandler


---


|  |  |  |
| --- | --- | --- |
| **Overview** \| **Package** \| **Class** \| **Tree** \| **Deprecated** \| **Index** \| **Help** \| **Weka's home** | |  |
| **PREV CLASS**   **NEXT CLASS** | **FRAMES**    **NO FRAMES**     **All Classes** |  |
| SUMMARY: NESTED \| FIELD \| CONSTR \| METHOD | DETAIL: FIELD \| CONSTR \| METHOD |  |


---


## weka.attributeSelection Interface StartSetHandler

**All Known Implementing Classes:**: BestFirst, GeneticSearch, GreedyStepwise, LinearForwardSelection, RandomSearch, Ranker

---

``` public interface StartSetHandler ```

Interface for search methods capable of doing something sensible
given a starting set of attributes.

**Version:**
:   $Revision: 1.8 $

**Author:**
:   Mark Hall (mhall@cs.waikato.ac.nz)

---

| **Method Summary** | |
| --- | --- |
| `java.lang.String` | `getStartSet()`             Returns a list of attributes (and or attribute ranges) as a String |
| `void` | `setStartSet(java.lang.String startSet)`             Sets a starting set of attributes for the search. |

**Method Detail**

### setStartSet

```
void setStartSet(java.lang.String startSet)
                 throws java.lang.Exception
```

:   Sets a starting set of attributes for the search. It is the
    search method's responsibility to report this start set (if any)
    in its toString() method.

    :   **Parameters:**: `startSet` - a string containing a list of attributes (and or ranges), eg. 1,2,6,10-15. **Throws:**: `java.lang.Exception` - if start set can't be set.

---


### getStartSet

```
java.lang.String getStartSet()
```

:   Returns a list of attributes (and or attribute ranges) as a String

    :   **Returns:**: a list of attributes (and or attribute ranges)


---


|  |  |  |
| --- | --- | --- |
| **Overview** \| **Package** \| **Class** \| **Tree** \| **Deprecated** \| **Index** \| **Help** \| **Weka's home** | |  |
| **PREV CLASS**   **NEXT CLASS** | **FRAMES**    **NO FRAMES**     **All Classes** |  |
| SUMMARY: NESTED \| FIELD \| CONSTR \| METHOD | DETAIL: FIELD \| CONSTR \| METHOD |  |


---
